# Synthesis of *γ*-pyrones via decarboxylative condensation of *β*-ketoacids

**DOI:** 10.1007/s00706-016-1851-2

**Published:** 2016-10-27

**Authors:** Jérémy Merad, Thomas Maier, Catarina A. B. Rodrigues, Nuno Maulide

**Affiliations:** Fakultät für Chemie, Institut für Organische Chemie, Universität Wien, Währinger Strasse 38, 1090 Vienna, Austria

**Keywords:** Heterocyclic chemistry, *γ*-Pyrones, Decarboxylative condensation, *β*-Ketoacids, Electrophilic activation, Convergent synthesis

## Abstract

**Abstract:**

This manuscript describes the convergent synthesis of aryl- and alkyl-disubstituted *γ*-pyrones from *β*-ketoacids. The reaction proceeds in the presence of trifluoromethanesulfonic anhydride via an unprecedented decarboxylative auto-condensation of the starting material. Herein, the scope and limitations of this transformation are reported.

**Graphical abstract:**

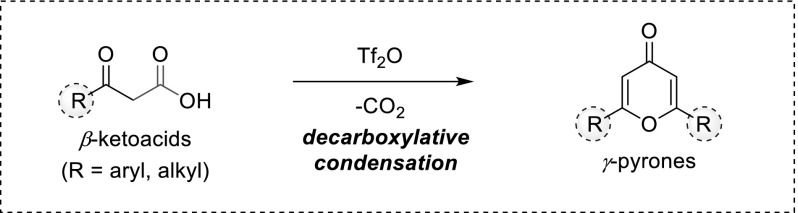

## Introduction

Since their discovery in the first part of the 19th century [1], heterocyclic derivatives occupy a special place in the field of organic chemistry. Indeed, they offer an infinite diversity of structures and often ensure the biological activity of the entire molecule. It is then not surprising that the development of heterocyclic chemistry remains an endless source of interest for organic practitioners [2].

**Fig. 1:**
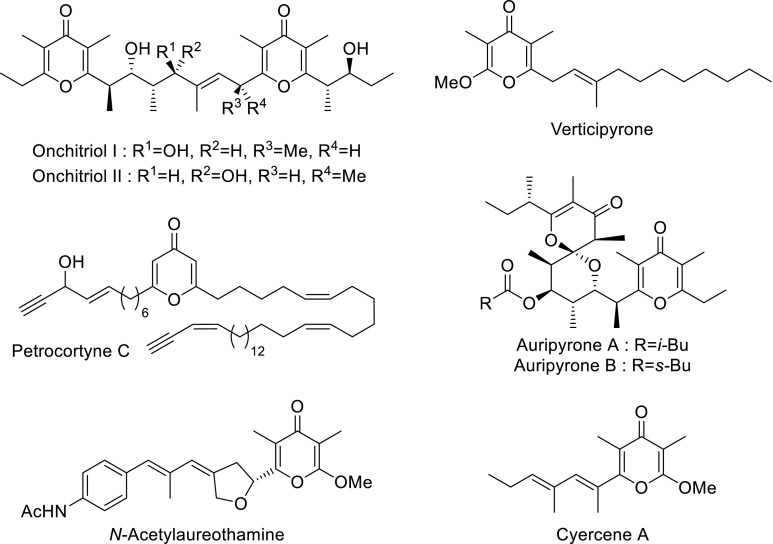
Examples of natural products incorporating a *γ*-pyrone moiety

In this context, pyrones constitute an essential class of unsaturated oxygenated six-membered heterocycles. As a consequence of their lactonic structure, *α*-pyrones represent highly valuable building blocks and a rich chemistry aiming their preparation has been developed [3]. Their vinologous isomers, *γ*-pyrones, are also involved in many fields of molecular sciences. Such moieties have been identified in the structure of numerous natural products [4, 5], such as the onchitriols I and II [6], petrocortyne C [7], cyercene A [8], verticipyrone [9], the auripyrones A and B [10], or *N*-acetylaureothamine [11], as but a small sample (Fig. 1). Due to their fully conjugated structure, some *γ*-pyrones also exhibit interesting photosensitizing properties [12, 13].

This broad range of applications has motivated the development of several methodologies to access these valuable heterocycles. Among them, the deoxygenative cyclization of a synthetic equivalent of linear 1,3,5-triketones [14] is undoubtedly the most extensively applied strategy to prepare alkyl-substituted *γ*-pyrones. The only significant modifications are related to the substrate generation [15] or the nature of the leaving group involved in the cyclization step [16–18]. More recently, an elegant acid-catalyzed cyclization of diynones has also been described [19]. Despite undeniable synthetic efficiency, these approaches require harsh reaction conditions or the time-consuming preparation of elaborate substrates. Consequently, further efforts are still needed to develop simple and fast syntheses of *γ*-pyrones using readily available starting materials.

From a retrosynthetic point of view, the condensation of identical fragments emerges as an attractive and privileged disconnection. This approach was successfully applied by Moghaddam et al. in a microwave-assisted synthesis of alkyl-substituted *γ*-pyrones from anhydrides and carboxylic acids [20]. During our investigations aiming to develop the potential of trifluoromethanesulfonic anhydride (Tf_2_O) as an electrophilic-activating reagent of unsaturated C–O bonds [21–26], we discovered that *β*-ketoacids can undergo an auto-condensation to form disubstituted *γ*-pyrones. This simple transformation, which we wish to present herein, allows the obtention of alkyl- and aryl-substituted heterocycles.

## Results and discussion

### Preparation of β-ketoacids

*β*-Ketoacids are known to be thermolabile species that undergo rather fast decarboxylation, resulting in the formation of the corresponding ketone. Hence, the purification and the storage of such unstable compounds are a difficult task usually discouraging their use as synthetic building blocks. Despite this undesirable property, we speculated that a procedure involving the efficient preparation of *β*-ketoacids from readily available *β*-ketoesters, their isolation by simple extraction, and their immediate engagement in the subsequent condensation might constitute a synthetically convenient approach.

The saponification of ethyl 3-oxo-3-phenylpropanoate proceeds smoothly when performed in the presence of an aqueous solution of NaOH at room temperature over 10 h (Scheme 1). Successive liquid–liquid extractions and a pH adjustment allowed the isolation of 3-oxo-3-phenylpropanoic acid (**1a**) from the unreacted starting material and salts. It should be noticed that, through this procedure, the products are systematically obtained in mixture with various amount of *decarboxylated* ketone. However, the yield in desired ketoacid is readily determined via ^1^H NMR analysis calculating the integration ratios between protons H^*b*^ and H^*c*^ (Scheme 1).

**Figure:**
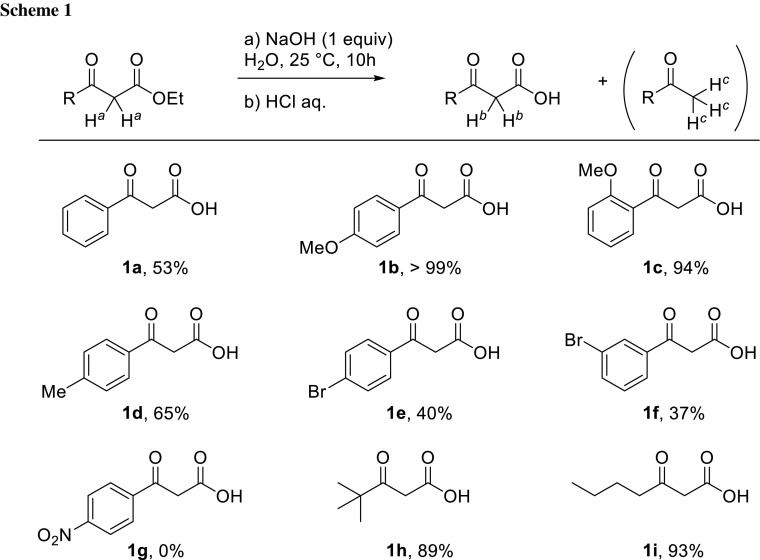

The reaction conditions tolerate diverse substituents on the aromatic ring, such as methoxy (**1b**, **1c**), methyl (**1d**), or halides (**1e**, **1f**). Alkyl ketoacids (**1h**, **1i**) are also obtained in very good yields. However, in the presence of a nitro substituent, the saponification does not occur and the starting material is recovered (**1g**). This last result is in line with the trend towards higher yields with increasing electron-donating properties of the R group (better yields obtained for R = alkyl or electron-rich aromatic ring). That observation can be rationalised by the higher acidity of H^*a*^ protons (Scheme 1) for electron-poor ketoesters, presumably enabling deprotonation—rather than saponification—by NaOH. Unfortunately, an excess of sodium hydroxide only leads to the isolation of degradation products.

### Synthesis of γ-pyrones via decarboxylative condensation

Tf_2_O is a highly electrophilic species able to polarise unsaturated C–O bonds and initiate domino transformations. In this way, a stoichiometric amount of Tf_2_O triggers the condensation of freshly prepared *β*-ketoacid **1a** to afford 2,6-diphenyl-4*H*-pyran-4-one (**2a**) in an 81% yield (Scheme 2). The same reactivity is observed for all the acids previously formed. Indeed, aromatic compounds with electron-donating substituents (**1b**–**1d**) appear to be suitable substrates and are readily converted into the corresponding heterocycles (**2b**–**2d**) with moderate-to-good yields. Decreasing the electron density on the aromatic ring results in lower yields (**2e**, **2f**). This might be explained by the higher instability of the corresponding *β*-ketoacids which undergo decarboxylation prior to the condensation. Branched alkyl substituents are well tolerated, and 2,6-di-*tert*-butyl-4*H*-pyran-4-one (**2h)** was obtained in a good yield. Due to the presence of multiple enolisable positions, the condensation of **1i** is accompanied by side reactions, but 2,6-dibutyl-4*H*-pyran-4-one (**2i**) was still isolated in a 10% yield.

**Figure:**
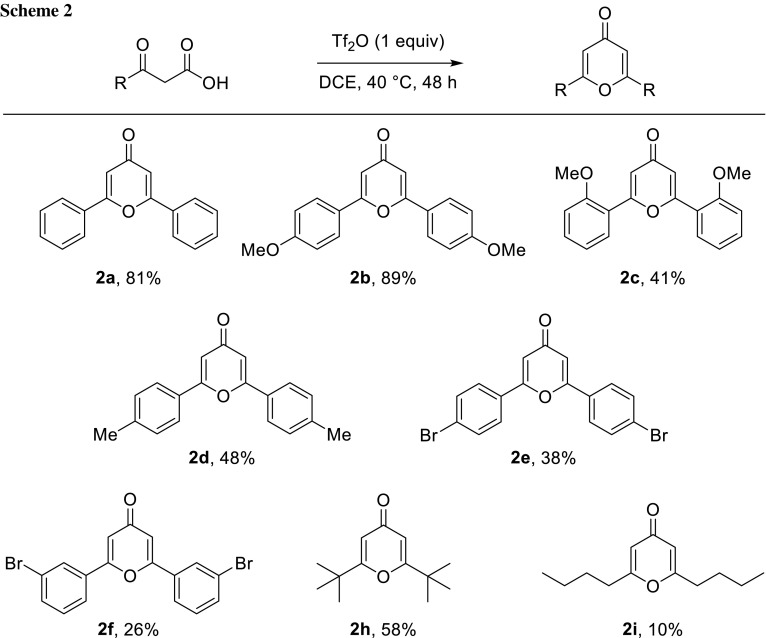

The Tf_2_O-triggered auto-condensation of *β*-ketoacids provides 2,6-disubstituted *γ*-pyrones through an in situ decarboxylation process. Mechanistic studies on this reaction are underway and shall be reported in due course.

## Conclusion

In summary, we report herein that *β*-ketoacids undergo an auto-condensation process when activated in the presence of Tf_2_O. Although unstable, such compounds can be easily prepared and constitute readily available substrates in the *decarboxylative* synthesis of 2,6-disubstituted *γ*-pyrones. This reaction tolerates aryl- as well as alkyl-derivatives, opening up an attractive and convergent way to the construction of these heterocycles.

## Experiment

Saponification reactions did not require the use of oven-dried glassware and were performed under air atmosphere. Auto-condensations of *β*-ketoacids were carried out under argon atmosphere using oven-dried glassware and freshly distillated trifluoromethanesulfonic anhydride. All other reagents and anhydrous solvents were used as received from commercial suppliers unless otherwise stated. Reaction progress was monitored by thin-layer chromatography (TLC) performed on plastic plates coated with kieselgel F254 with 0.2 mm thickness or GC–MS. Visualization was achieved by ultraviolet light (254 nm) or development with KMnO_4_ solution. Flash column chromatography was performed using silica gel 60 (230–400 mesh, Merck and co.). Near infrared spectra were recorded using a Perkin-Elmer Spectrum 100 FT-IR spectrometer. Mass spectra were obtained using a Finnigan MAT 8200 or (70 eV) or an Agilent 5973 (70 eV) spectrometer, using electrospray ionization (ESI). All ^1^H NMR, ^13^C NMR spectra were recorded on Bruker AV-400 in CDCl_3_ or *d*_*6*_-DMSO. Chemical shifts are given in parts per million (*δ*/ppm).

### General procedure for the synthesis of γ-pyrones from β-ketoesters

*β*-Ketoester (5 mmol) was added onto an aqueous solution (5 cm^3^) of NaOH (1 equiv, 5 mmol, 200 mg). The mixture was stirred under air atmosphere at room temperature over 12 h. Et_2_O (5 cm^3^) was then added. The layers were separated, and the aqueous one was washed with Et_2_O (2 × 5 cm^3^) before to be acidified with a concentrated solution of HCl (37% w:w in H_2_O) to reach pH 1. The product was then extracted with EtOAc (3 × 5 cm^3^). The combined organic layers were dried onto anhydrous Na_2_SO_4_, filtered, and concentrated under reduced pressure to give the desired *β*-ketoacid in mixture with the corresponding decarboxylated ketone. The yield in *β*-ketoacid was determined by ^1^H NMR experiments integrating characteristic signals of *β*-ketoacid (and its enol tautomer) and ketone. Then, a part of the mixture was directly engaged in the subsequent condensation reaction.

*β*-ketoacid (1 equiv) was added portionwise onto a solution of Tf_2_O (1 equiv) in dichloroethane (0.5 M) under argon atmosphere at 40 °C, and the mixture was stirred at this temperature over 2 days. After cooling at room temperature, a saturated solution of Na_2_CO_3_ was added and the layers were separated. The aqueous one was then extracted with dichloromethane (3 x), and the combined organic layers were dried with anhydrous Na_2_SO_4_, filtered, and concentrated under vacuum. The purification of the crude material by silica gel column chromatography provided the desired *γ*-pyrone derivative.

#### *2,6*-*Diphenyl*-*4H*-*pyran*-*4*-*one* (**2a**)

The product was obtained starting from 0.5 mmol of *β*-ketoacid [determined by ^1^H NMR (400 MHz, *d*_6_-DMSO) using the characteristic signals: *δ* = 4.05 (s, 2H, CH_2_ *β*-ketoacid), 2.58 (s, 3H, CH_3_ ketone)]. Yellowish solid (50 mg, 0.20 mmol, 81%) after purification by column chromatography (heptane/EtOAc = 60/40). ^1^H NMR (400 MHz, CDCl_3_): *δ* = 7.90-7.81 (m, 4H), 7.57-7.50 (m, 6H), 6.82 (s, 2H) ppm; ^13^C NMR (101 MHz, CDCl_3_): *δ* = 180.4, 163.5, 131.7, 131.6, 129.3, 126.1, 111.6 ppm; IR: $\bar{\nu }$ = 1645.33, 1606.41, 1572.92, 1493.18, 1450.50, 1390.51, 946.76, 864.75, 772.19, 688.69 cm^−1^. Physical data are in accordance with the literature [19].

#### *2,6*-*Bis(4*-*methoxyphenyl)*-*4H*-*pyran*-*4*-*one* (**2b**, C_19_H_16_O_4_)

The product was obtained starting from 0.36 mmol of *β*-ketoacid [determined by ^1^H NMR (400 MHz, *d*_6_-DMSO) using the characteristic signals: *δ* = 3.97 (s, 2H, CH_2_ *β*-ketoacid), 2.57 (s, 3H, CH_3_ ketone)]. White solid (49 mg, 0.16 mmol, 89%) after purification by column chromatography (heptane/EtOAc = 40/60). ^1^H NMR (400 MHz, CDCl_3_): *δ* = 7.79 (d, ^*3*^ *J* = 8.9 Hz, 4H), 7.02 (d, ^*3*^ *J* = 8.9 Hz, 4H), 6.68 (s, 2H), 3.88 (s, 6H) ppm; ^13^C NMR (101 MHz, CDCl_3_): *δ* = 180.5, 163.3, 162.3, 127.7, 124.1, 114.7, 109.9, 55.6 ppm; IR: $\bar{\nu }$ = 3413.34, 3073.07, 2929.16, 1842.10, 1642.00, 1602.30, 1507.76, 1461.64, 1425.15, 1388.20, 1259.26, 1177.43, 1022.74, 832.33 cm^−1^; HRMS (ESI): *m/z* calculated for [M+H]^+^ 309.1121, found 309.1108.

#### *2,6*-*Bis(2*-*methoxyphenyl)*-*4H*-*pyran*-*4*-*one* (**2c**, C_19_H_16_O_4_)

The product was obtained starting from 0.29 mmol of *β*-ketoacid [determined by ^1^H NMR (400 MHz, *d*_6_-DMSO) using the characteristic signals: *δ* = 3.85 (s, 2H, CH_2_ *β*-ketoacid), 5.95 (s, 1H, CH enol tautomer), 2.52 (s, 3H, CH_3_ ketone)]. White solid (18 mg, 0.058 mmol, 41%) after purification by column chromatography (heptane/EtOAc = 40/60). ^1^H NMR (400 MHz, CDCl_3_): *δ* = 7.79 (dd, ^*3*^ *J* = 7.8 Hz, ^*4*^ *J* = 1.7 Hz, 2H), 7.46 (ddd, ^*3*^ *J* = 8.4, 7.8 Hz, ^*4*^ *J* = 1.8 Hz, 2H), 7.09 (s, 2H), 7.08 (ddd, ^*3*^ *J* = 7.8, 7.8 Hz, ^*4*^ *J* = 1.0 Hz, 2H), 7.04 (dd, ^*3*^ *J* = 8.4 Hz, ^*4*^ *J* = 1.0 Hz, 2H), 3.94 (s, 6H) ppm; ^13^C NMR (101 MHz, CDCl_3_): *δ* = 181.4, 161.2, 157.9, 132.2, 129.3, 121.0, 120.9, 116.1, 111.9, 55.8, 29.8 ppm; IR: $\bar{\nu }$ = 3413.45, 3078.18, 2924.97, 2849.82, 1637.68, 1588.26, 1491.72, 1458.57, 1392.75, 1293.53, 1250.97, 1019.97, 757.63 cm^−1^; HRMS (ESI): *m/z* calculated for [M+H]^+^ 309.1121, found 309.1108.

#### *2,6*-*Di*-*(p*-*tolyl)*-*4H*-*pyran*-*4*-*one* (**2d**)

The product was obtained starting from 3.14 mmol of *β*-ketoacid [determined by ^1^H NMR (400 MHz, CDCl_3_) using the characteristic signals: *δ* = 4.05 (s, 2H, CH_2_ *β*-ketoacid), 5.68 (s, 1H, CH enol tautomer), 2.58 (s, 3H, CH_3_ ketone)]. Yellow solid (210 mg, 0.76 mmol, 48%) after purification by column chromatography (heptane/EtOAc = 60/40). ^1^H NMR (400 MHz, CDCl_3_): *δ* = 7.73 (d, ^*3*^ *J* = 8.3 Hz, 4H), 7.32 (d, ^*3*^ *J* = 8.0 Hz, 4H), 6.75 (s, 2H), 2.43 (s, 6H) ppm; ^13^C NMR (101 MHz, CDCl_3_): *δ* = 180.5, 163.5, 142.0, 130.0, 128.9, 126.0, 110.8, 21.6 ppm; IR: $\bar{\nu }$ = 3363.26, 3067.44, 2920.18, 2852.73, 1643.46, 1607.22, 1508.89, 1413.77, 1384.45, 943.62, 819.27 cm^−1^. Physical data are in accordance with the literature [19].

#### *2,6*-*Bis(4*-*bromophenyl)*-*4H*-*pyran*-*4*-*one* (**2e**, C_17_H_10_Br_2_O_2_)

The product was obtained starting from 0.25 mmol of *β*-ketoacid [determined by ^1^H NMR (400 MHz, *d*_6_-DMSO) using the characteristic signals: *δ* = 4.04 (s, 2H, CH_2_ *β*-ketoacid), 5.85 (s, 1H, CH enol tautomer), 2.56 (s, 3H, CH_3_ ketone)]. White solid (19 mg, 0.047 mmol, 38%) after purification by column chromatography (heptane/EtOAc = 60/40). ^1^H NMR (400 MHz, CDCl_3_): *δ* = 7.79-7.54 (m, 8H), 6.79 (s, 2H) ppm; ^13^C NMR (101 MHz, CDCl_3_): *δ* = 179.8, 162.6, 132.7, 130.5, 127.5, 126.4, 111.9 ppm; IR: $\bar{\nu }$ = 3365.83, 3051.06, 2922.62, 1652.22, 1614.11, 1486.45, 1410.83, 1379.67, 946.70, 821.47 cm^−1^; HRMS (ESI): *m/z* calculated for [M+H]^+^ 404.9120, found 404.9122.

#### *2,6*-*Bis(3*-*bromophenyl)*-*4H*-*pyran*-*4*-*one* (**2f**, C_17_H_10_Br_2_O_2_)

The product was obtained starting from 4.16 mmol of *β*-ketoacid [determined by ^1^H NMR (400 MHz, *d*_6_-DMSO) using the characteristic signals: *δ* = 4.08 (s, 2H, CH_2_ *β*-ketoacid), 5.89 (s, 1H, CH enol tautomer), 2.59 (s, 3H, CH_3_ ketone)]. White solid (21 mg, 0.052 mmol, 26%) after purification by column chromatography (heptane/EtOAc = 60/40). ^1^H NMR (400 MHz, CDCl_3_): *δ* = 7.96 (dd, ^*4*^ *J* = 1.7, 1.7 Hz, 2H), 7.76 (ddd, ^*3*^ *J* = 7.9 Hz, ^*4*^ *J* = 1.7, 1.0 Hz, 2H), 7.68 (ddd, ^*3*^ *J* = 7.9 Hz, ^*4*^ *J* = 1.7, 1.0 Hz, 2H), 7.42 (dd, ^*3*^ *J* = 7.9, 7.9 Hz, 2H), 6.78 (s, 2H) ppm; ^13^C NMR (101 MHz, CDCl_3_): *δ* = 179.6, 162.1, 134.6, 133.5, 130.9, 129.1, 124.7, 123.5, 112.5 ppm; IR: $\bar{\nu }$ = 3362.24, 3081.69, 2922.50, 2852.62, 1656.61, 1616.13, 1582.05, 1424.06, 1379.87, 1256.10, 1230.82, 917.37, 872.12, 785.53, 687.67 cm^−1^; HRMS (ESI): *m/z* calculated for [M+H]^+^ 404.9120, found 404.9101.

#### *2,6*-*Di*-*(tert*-*butyl)*-*4H*-*pyran*-*4*-*one* (**2g**)

The product was obtained starting from 0.39 mmol of *β*-ketoacid [determined by ^1^H NMR (400 MHz, CDCl_3_) using the characteristic signals: *δ* = 3.60 (s, 2H, CH_2_ *β*-ketoacid), 5.10 (s, 1H, CH enol tautomer), 2.14 (s, 3H, CH_3_ ketone)]. White solid (250 mg, 1.2 mmol, 58%) after purification by column chromatography (heptane/EtOAc = 30/70). ^1^H NMR (400 MHz, CDCl_3_): *δ* = 6.11 (s, 2H), 1.26 (s, 18H) ppm; ^13^C NMR (101 MHz, CDCl_3_): *δ* = 181.5, 175.4, 109.6, 36.4, 27.9 ppm; IR: $\bar{\nu }$ = 3364.25, 2961.00, 2933.97, 2870.52, 1648.26, 1613.70, 1481.30, 1461.79, 1397.12, 1380.17, 1361.50, 1256.56, 1221.79, 941.68, 921.52, 873.82 cm^−1^. Physical data are in accordance with the literature [19].

#### *2,6*-*Dibutyl*-*4H*-*pyran*-*4*-*one* (**2h**, C_13_H_20_O_2_)

The product was obtained starting from 4.16 mmol of *β*-ketoacid [determined by ^1^H NMR (400 MHz, CDCl_3_) using the characteristic signals: *δ* = 3.50 (s, 2H, CH_2_ *β*-ketoacid), 5.02 (s, 1H, CH enol tautomer), 2.04 (s, 3H, CH_3_ ketone)]. Colorless oil (45 mg, 0.22 mmol, 10%) after purification by column chromatography (heptane/EtOAc = 50/50). ^1^H NMR (400 MHz, CDCl_3_): *δ* = 6.04 (s, 2H), 2.48 (t, ^*3*^ *J* = 7.5 Hz, 2H), 1.66–1.56 (m, 2H), 1.45–1.31 (m, 2H), 0.93 (t, ^*3*^ *J* = 7.3 Hz, 3H) ppm; ^13^C NMR (101 MHz, CDCl_3_): *δ* = 180.6, 169.3, 113.2, 33.3, 28.9, 22.1, 13.8 ppm; IR: $\bar{\nu }$ = 3432.25, 2957.44, 2930.25, 2871.43, 1660.81, 1618.39, 1464.48, 1396.42, 1147.33, 929.54, 863.93 cm^−1^; HRMS (ESI): *m/z* calculated for [M+H]^+^ 209.1536, found 209.1540.
